# Exercise Training Increases Parietal Lobe Cerebral Blood Flow in Chronic Stroke: An Observational Study

**DOI:** 10.3389/fnagi.2017.00318

**Published:** 2017-09-29

**Authors:** Andrew D. Robertson, Susan Marzolini, Laura E. Middleton, Vincenzo S. Basile, Paul I. Oh, Bradley J. MacIntosh

**Affiliations:** ^1^Heart and Stroke Foundation Canadian Partnership for Stroke Recovery, Ottawa, ON, Canada; ^2^Hurvitz Brain Sciences, Sunnybrook Research Institute, University of Toronto, Toronto, ON, Canada; ^3^Toronto Rehab, University Health Network, Toronto, ON, Canada; ^4^Department of Kinesiology, University of Waterloo, Waterloo, ON, Canada; ^5^Division of Neurology, Mackenzie Health, Richmond Hill, ON, Canada; ^6^Peter Munk Cardiac Centre, University of Toronto, Toronto, ON, Canada

**Keywords:** aerobic exercise, arterial spin labeling, cerebrovascular circulation, cerebrovascular disease, rehabilitation

## Abstract

Exercise is increasingly recommended as an essential component of stroke rehabilitation, yet uncertainty remains with respect to its direct effect on the cerebral vasculature. The current study first demonstrated the repeatability of pseudo-continuous arterial spin labeling (ASL) magnetic resonance imaging (MRI) in older adults with stroke, and then investigated the change in cerebrovascular function following a 6-month cardiovascular rehabilitation program. In the repeatability study, 12 participants at least 3 months post-stroke underwent two ASL imaging scans 1 month apart. In the prospective observational study, eight individuals underwent ASL imaging and aerobic fitness testing before and after a 6-month cardiovascular rehabilitation program. Cerebral blood flow (CBF) and the spatial coefficient of variation of CBF (sCoV) were quantified to characterize tissue-level perfusion and large cerebral artery transit time properties, respectively. In repeat scanning, intraclass correlation (ICC) indicated moderate test-retest reliability for global gray matter CBF (ICC = 0.73) and excellent reliability for sCoV (ICC = 0.94). In the observational study, gray matter CBF increased after training (baseline: 40 ± 13 vs. 6-month: 46 ± 12 ml·100 g^−1^·min^−1^, *P* = 0.036). The greatest change occurred in the parietal lobe (+18 ± 12%). Gray matter sCoV, however, did not change following training (*P* = 0.31). This study provides preliminary evidence that exercise-based rehabilitation in chronic stroke enhances tissue-level perfusion, without changing the relative hemodynamic properties of the large cerebral arteries.

## Introduction

Aerobic exercise training after stroke impacts functional recovery (Duncan et al., 2003) and cognition (Zheng et al., 2016). While these changes may be driven by improvements in cardiopulmonary fitness (Marsden et al., 2013) or cardiometabolic risk profile (Tang et al., 2014), less is known about the role of brain-specific effects. Training-related increases in regional cerebral blood flow (CBF), particularly within the anterior cingulate cortex, have been reported in healthy older adults and those with coronary artery disease (Chapman et al., 2013; Anazodo et al., 2015); yet conclusive evidence in people with cerebrovascular disease is lacking. One trial involving individuals with chronic stroke used transcranial Doppler ultrasound to evaluate exercise-based changes in cerebrovascular hemodynamics (Ivey et al., 2011). In that study, aerobic training was found to enhance cerebrovascular reactivity, but not resting mean blood flow velocity within the middle cerebral artery. More recently, a magnetic resonance imaging (MRI) study with chronic stroke participants reported that medial temporal lobe perfusion, but not global CBF, was increased following a community exercise program involving aerobic, resistance, flexibility and balance training (Moore et al., 2015).

Arterial spin labeling (ASL) MRI is a reliable and repeatable method for quantifying tissue level CBF in healthy older adults and those with mild cognitive impairment (Kilroy et al., 2014). The technique has also gained increasing support for its clinical utility in stroke patients (Zaharchuk, 2011; Guo et al., 2014), though repeatability in this population has not been demonstrated. In addition to altered perfusion, cerebrovascular disease can manifest as a change in the temporal dynamics of blood flow. Arterial transit time quantifies the time required for blood to pass from large arteries to the tissue, and prolonged transit time may be indicative of hemodynamic impairment independent of perfusion deficits (MacIntosh et al., 2010; Kamano et al., 2013). When transit time is prolonged, a proportion of the ASL signal remains in conduit arteries at the time of imaging and the signal intensity within the resulting CBF image becomes spatially varying relative to the distribution of blood through the large cerebral arteries. Thus, the spatial coefficient of variation of the CBF image across gray matter (sCoV) is an indirect measure of arterial transit time—an association which was previously validated in a multiple post-label delay ASL study involving hypertensive older adults (Mutsaerts et al., 2017). This preliminary study aimed to examine CBF and sCoV in older adults with chronic stroke following a clinical exercise rehabilitation program. Given that previous research in cohorts with vascular disease has not observed training effects on global CBF (Anazodo et al., 2015; Moore et al., 2015), we tested for regional effects within the 4 major cortical lobes. We hypothesized that exercise training would increase CBF and reduce sCoV, particularly within the frontal lobe. To demonstrate the validity our findings, we first assessed the repeatability of ASL CBF and sCoV in chronic stroke.

## Materials and Methods

### Participants

#### Repeatability Study

Individuals with chronic stroke were recruited from a local neurology clinic between April 2012 and February 2014. Eligible participants were at least 3 months post-stroke, able to ambulate at least 10 m with or without an assistive device, and did not present with stroke-related lower limb impairment, dementia, or contraindications to exercise testing or MRI. Although etiology of lacunar, ischemic and hemorrhagic stroke differ (Sierra et al., 2011), we chose not to limit recruitment by stroke type so as to examine a wider range of hemodynamic profiles. These participants underwent two MRI scans not more than 1 month apart.

#### Observational Study

A separate group of individuals with chronic stroke were recruited from a cardiovascular rehabilitation program designed to improve fitness, strength and cardiovascular risk. Consecutive participants were recruited between September 2013 and November 2014 using the same eligibility criteria as above. Recruitment occurred after referral to the clinical rehabilitation program; hence, randomization was not feasible. Participants in the rehabilitation program performed cardiopulmonary assessments at baseline, 3-month and 6-month time points; and underwent MRI at baseline and 6-month time points. The protocols from both the repeatability and observational studies were approved by and carried out in accordance with recommendations from the Sunnybrook Research Ethics Board and the University Health Network Research Ethics Board. All participants gave written informed consent in accordance with the Declaration of Helsinki.

### MRI Acquisition

We acquired neuroimaging using a 3-Tesla MRI system (Achieva, Philips Healthcare, Best NL) with a body coil transmitter and an eight-channel head coil receiver. All imaging was acquired at least 24 h after exercise. Structural imaging included T1-weighted (TR/TE = 9.5/2.3 ms, flip angle = 8°, voxel dimensions = 0.9 × 0.7 × 1.2 mm^3^, field of view (FOV) = 240 × 191 × 168 mm^3^) and fluid-attenuated inversion recovery (TR/TE/TI = 9000/125/2800 ms, flip angle = 90°, voxel dimensions = 0.4 × 0.4 × 3 mm^3^, FOV = 240 × 240 × 156 mm^3^) acquisitions. Perfusion imaging involved a pseudo-continuous ASL acquisition. A train of radio frequency pulses (duration = 0.5 ms, flip angle = 18°, interpulse pause = 0.5 ms) with a balanced gradient scheme was applied over a label duration of 1650 ms. The labeling plane was prescribed perpendicular to the internal carotid artery, at least 5 mm distal to the carotid bifurcation as visualized by a phase-contrast scout image. Thirty control and tag volume pairs were acquired by multi-slice 2D echo planar imaging (TR/TE = 4000/9.6 ms, flip angle = 90°, in-plane resolution = 3 × 3 mm^3^, FOV = 192 × 192 mm^3^, slice thickness = 5 mm, number of slices = 18 (no gap), post-label delay = 1600 ms first slice and ascending for subsequent slices). The ASL FOV limited acquisition of the inferior border of the temporal lobes and superior border of the frontal lobes in some participants. A proton density-weighted reference volume was acquired to estimate the equilibrium magnetization and extract a receiver coil sensitivity profile (TR = 10 s, but otherwise identical to ASL parameters).

### MRI Processing

We used the fMRIB Software Library (package fsl-4.1)^1^ for processing. Brain extraction (Smith, 2002) and segmentation (Zhang et al., 2001) tools isolated gray matter, white matter and cerebrospinal fluid from the T1-weighted image. Ischemic lesion volume was identified by a trained analyst (ADR) using hypointense segmentation of the T1-weighted image, manual editing and confirmation against the fluid-attenuated inversion recovery image. CBF was calculated from the ASL control-tag difference images after motion correction (Shirzadi et al., 2015) and spatial smoothing with a 5-mm Gaussian kernel. CBF levels were adjusted slice-by-slice to account for the incremental post-label delay, and calibrated by a proton density-weighted image (Alsop et al., 2015). CBF estimates were restricted to gray matter and did not include the ischemic lesion. CBF within the stroke lesion was assessed separately. Spatial CoV was calculated by dividing the standard deviation of gray matter CBF by the mean and expressed as a percentage. CBF and sCoV were averaged across all gray matter voxels for the whole brain analysis, and across all gray matter voxels within four regions of interest (ROI) comprising the major cortical lobes (right and left hemispheres combined), as defined by the Montreal Neurological Institute atlas, for the regional analysis.

### Rehabilitation Program

For the observational study, exercise training occurred within a common model of care for cardiac rehabilitation (Hamm and Kavanagh, 2000). It involved aerobic and resistance training, as well as lifestyle education over 6 months, and was supervised by Cardiac Rehabilitation Supervisors. This model is consistent with consensus guidelines for exercise in stroke survivors recommended by the American Heart Association and American Stroke Association (Billinger et al., 2014). The aerobic component involved one supervised session and four independent sessions per week. The mode of exercise was flexible around individual preferences to optimize adherence. The initial aerobic prescription was set at 20 min of moderate intensity exercise (60%–80% of oxygen uptake reserve), based on the baseline symptom-limited cardiopulmonary assessment. The duration of exercise was gradually progressed up to a maximum of 60 min per session. The 3-month cardiopulmonary assessment, diary information and patient feedback to the rehabilitation supervisor guided the progression of exercise intensity. Independent aerobic exercise was tracked with a diary, noting the mode, duration and heart rate of each session. Strength training targeting the upper body (five exercises), lower body (three exercises) and core (two exercises) was initiated 2 months into the program using hand-held dumbbells, body weight and elastic bands for resistance. Following a series of supervised sessions to demonstrate proper technique, participants were asked to complete two independent sessions per week through the remainder of the program. MRI at 6 months occurred within 2 weeks of completing the rehabilitation program.

### Cardiopulmonary Assessment

Participants completed symptom-limited graded exercise tests on an upright cycle ergometer at baseline, 3 months and 6 months. Work load was increased by either 8.2 or 16.3 W/min, maintaining a pedaling rate of 60 rpm. The lower rate of progression was used for one participant who exhibited low functional ability at screening. Breath-by-breath gases were sampled to quantify peak oxygen uptake ($\overset{\cdot }{\text{V}}{\text{O}}_{2}\text{peak}$) and heart rate was monitored continuously. The test was discontinued at participant request, or if they were unable to maintain the required cadence, displayed adverse clinical signs or symptoms, or reached a physiological maximum (Marzolini et al., 2012).

### Statistical Analysis

We performed statistical analyses using R (version 3.3.1)^2^. Unless otherwise noted, all analyses used R’s standard set of libraries. Data are reported as mean ± standard deviation, with *P* < 0.05 interpreted as significant. Group differences between the repeatability sample and the observational sample were examined by unpaired *t*-test and Fisher’s exact test for continuous and categorical variables, respectively. In the repeatability study, we used intraclass correlation (ICC) and within-subject coefficient of variation to assess test-retest reliability. ICC estimates (95% confidence intervals) were calculated using a two-way mixed-effects model based on a single measurement-absolute agreement approach (R Library: irr). Gray matter CBF and sCoV repeatability were also assessed by evaluating the mean interscan difference, the correlation between interscan difference and interscan mean, and the repeatability coefficient (Bland and Altman, 1986). In the observational study, a mixed-model approach with random intercept and slope was used to assess the effect of training on aerobic fitness (R libraries: lme4 and multcomp). This approach was used to account for missing data from four participants who chose not to complete the 6-month assessment for personal reasons. Baseline $\overset{\cdot }{\text{V}}{\text{O}}_{2}\text{peak}$ was not different between participants that completed the 6-month assessment and those that did not, so all participants were retained for the 6-month cerebrovascular analysis. Paired *t*-tests compared whole brain gray matter CBF and sCoV between the baseline and 6-month time points. In the regional analysis, a repeated-measures ANOVA model tested for an interaction between time and region of interest. *Post hoc* pairwise comparisons between baseline and 6-month CBF for each lobe were corrected by Bonferroni adjustment. Given the small sample, estimated effect sizes for the impact of training on gray matter CBF and sCoV were calculated using Hedges’ g for paired samples (Gibbons et al., 1993; R Library: effsize). Using these effect sizes, sample size calculations to inform future work were generated based on an ANOVA method with *α* = 0.05, Power = 0.8 and a correlation among repeated measures of 0.5 (G*Power software v.3.0.10)^3^.

## Results

Fourteen individuals were enrolled in the repeatability study. Two participants withdrew (non-contact, time commitment) prior to the second MRI, leaving 12 participants for analysis. Fourteen individuals were enrolled in the observational study. Six participants withdrew prior to the end of the rehabilitation program for the following reasons: medical—unrelated to the intervention (*n* = 4) and time commitment (*n* = 2). Participants from each study matched well for age and sex. Most participants had ischemic stroke; however, the repeatability study included one case of hemorrhagic stroke and one case of lacunar stroke, and the observational study included one case of lacunar stroke. Neither the baseline cerebrovascular characteristics nor the response to training of these individuals was distinctive from those with ischemic stroke. Participants in the repeatability study were further along post-stroke (Table 1).

**Table 1:** Participant demographics and clinical characteristics.

	Repeatability sample	Observational sample	*P* value
*N*	12	8	
Age, years	64 ± 16	67 ± 11	0.70
Sex—female, *n* (%)	4 (33)	2 (25)	1.00
Body mass index, kg·m^−2^	26 ± 4	27 ± 5	0.43
Peak oxygen uptake, ml·kg^−1^·min^−1^	22.1 ± 6.6	17.2 ± 5.8	0.100
Hypertension, *n* (%)	9 (75)	7 (88)	0.62
Dyslipidemia, *n* (%)	10 (83)	3 (38)	0.062
Diabetes mellitus, *n* (%)	5 (42)	1 (12)	0.32
Stroke characteristics			
Type			1.00
Hemorrhagic, *n* (%)	1 (8)	0 (0)	
Ischemic, *n* (%)	10 (83)	7 (88)	
Lacunar, *n* (%)	1 (8)	1 (12)	
Region			0.97
Cortical, *n* (%)	5 (42)	4 (50)	
Subcortical, *n* (%)	3 (25)	2 (25)	
White matter, *n* (%)	1 (8)	0 (0)	
Subtentorial, *n* (%)	3 (25)	2 (25)	
Hemisphere—right, *n* (%)	3 (25)	3 (38)	0.64
Time from stroke, months	20 ± 17	5 ± 3	0.020
Lesion volume, %	0.3 ± 0.3	0.4 ± 0.8	0.76

*Mean ± SD or count (percentage). P value reflects main effect of group*.

### Repeatability

**Figure 1:**
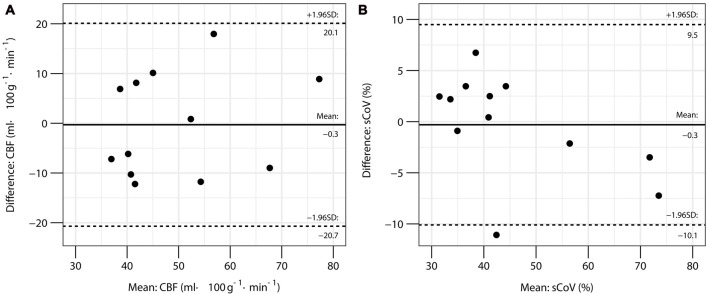
Bland-Altman plots for global gray matter cerebral blood flow (CBF) **(A)** and spatial coefficient of variation (sCoV) **(B)** in the repeatability study.

From the first scan, whole brain gray matter CBF was 51 ± 15 ml·100 g^−1^·min^−1^ and sCoV was 45 ± 12%. Gray matter CBF at the 2 time points had a mean interscan difference of −0.3 ml·100 g^−1^·min^−1^. No correlation between the individual interscan differences and means was observed (*r* = 0.22, *P* = 0.50) indicating that the difference between scans was stable over the range of CBF values, although the distribution of differences was wide with a repeatability coefficient of 20.4 ml·100 g^−1^·min^−1^ (Figure 1A). Gray matter sCoV had a mean interscan difference of −0.3% and the repeatability coefficient was 9.8%, suggesting a tighter distribution of differences compared to CBF. A trend for a correlation between individual differences and means was noted (*r* = −0.55, *P* = 0.066; Figure 1B).

ICC indicated moderate test-retest reliability for global gray matter CBF, but wide confidence intervals were noted (0.73; 95%CI: 0.28, 0.92). In contrast, the sCoV metric had good-to-excellent repeatability (0.94, 95%CI: 0.82, 0.98). Similarly, the within-subject coefficient of variation for sCoV suggested low variability between tests (6.1%; 95%CI: 3.0, 9.2); whereas, global CBF exhibited about double the test-retest variability (13.4%; 95%CI: 9.8, 17.2). Regional analysis showed repeatability was greatest in the frontal lobes for both CBF and sCoV (Table 2).

**Table 2:** Reliability metrics for regional gray matter cerebral blood flow (CBF) and spatial coefficient of variation (sCoV).

	ws-Coefficient of variation (95% CI)		Intraclass correlation (95% CI)	
	CBF	sCoV	CBF	sCoV
Frontal lobe	12.2 (8.2, 16.1)	6.6 (2.4, 10.8)	0.80 (0.45, 0.94)	0.93 (0.77, 0.98)
Occipital lobe	15.7 (10.4, 20.9)	11.3 (6.4, 16.3)	0.67 (0.20, 0.89)	0.90 (0.72, 0.97)
Parietal lobe	14.9 (10.3, 19.5)	8.3 (4.6, 12)	0.70 (0.26, 0.90)	0.92 (0.76, 0.97)
Temporal lobe	13.4 (9.6, 17.1)	9.4 (2.7, 16.0)	0.64 (0.14, 0.87)	0.76 (0.38, 0.92)

### Training

Participants in the rehabilitation program attended 76 ± 22% of supervised sessions and completed journal entries for aerobic exercise on 81 ± 29 days (i.e., ~68% of the targeted aerobic sessions). Self-reported exercise duration was 119 ± 22 min per week during the first month of the program, and progressed to 157 ± 34 min per week over the final month. The preferred modes of aerobic exercise in increasing order of frequency were treadmill walking, stationary cycling and over-ground walking. $\overset{\cdot }{\text{V}}{\text{O}}_{2}\text{peak}$ increased 28% between the baseline and 3-month assessments, and was stable between 3 months and 6 months. Patients expended the same relative effort at each test as peak respiratory exchange ratio was consistent across tests (Table 3).

**Table 3:** Cardiopulmonary assessment results.

	Baseline	3-Month	6-Month^†^	*P* value
Duration, min	5.4 ± 1.6	6.5 ± 1.2*	5.9 ± 1.9*	0.016
Respiratory exchange ratio	1.10 ± 0.07	1.13 ± 0.09	1.15 ± 0.10	0.38
Peak oxygen uptake, ml·kg^−1^·min^−1^	17.2 ± 5.8	22.1 ± 5.2*	20.7 ± 7.7	0.021

Mean ± SD. P value reflects main effect of time. ^†^Only four participants completed the 6-month assessment. *Difference from Baseline, P_Bonferroni_ < 0.05.

### Cerebral Hemodynamics and Exercise Training

**Figure 2:**
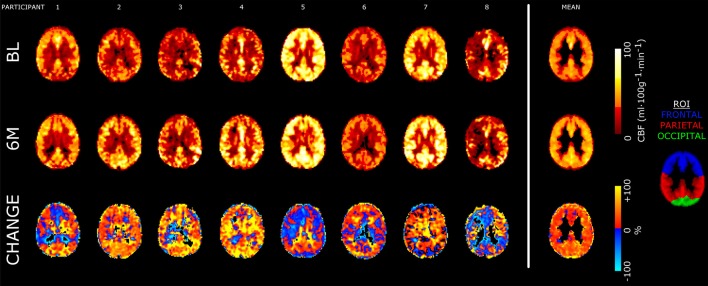
Axial slices of cerebral blood flow (CBF) images from each participant as well as the group mean from the observational study. CBF maps for baseline (BL) and 6-month (6M) scans are shown in absolute units (ml·100 g^−1^·min^−1^). The bottom row shows relative percentage change in CBF following training. Slices are taken near the superior edge of the lateral ventricles (*z* = 24 mm in the Montreal Neurological Institute atlas), showing portions of the frontal, parietal and occipital regions of interest (ROI). All images are registered to 3-mm isotropic voxels. Mean images are masked to gray matter ROI.

**Figure 3:**
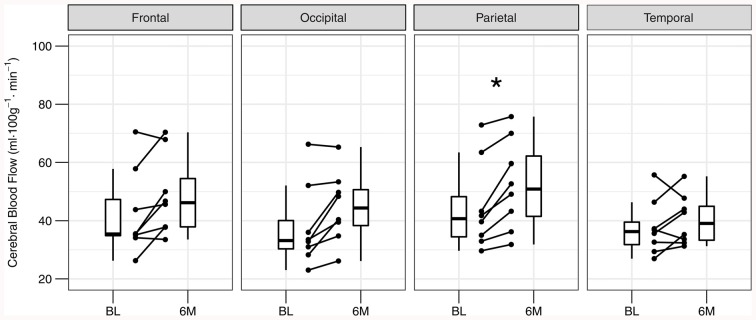
Individual changes in regional gray matter cerebral blood flow at baseline (BL) and after 6 months (6M) of exercise training. Post-training cerebral blood flow is elevated in the parietal lobe after correcting for multiple comparisons. *P_Bonferroni_ < 0.05.

Global gray matter CBF increased 15% at 6 months compared to baseline (baseline: 40 ± 13; 6-month: 46 ± 12 ml·100 g^−1^·min^−1^; *P* = 0.036). The effect size (Hedge’s g (95% confidence interval)) for the change in global gray matter CBF with exercise training was 0.27 (−0.80, 1.35). Figure 2 shows axial views from baseline and 6-month CBF maps for each participant in the training group. A time-by-region of interest interaction effect on CBF was noted (*P* = 0.022), in which the largest effect occurred in the parietal lobe (18 ± 12%; Figure 3). The parietal lobe was the only ROI in which all participants showed an increase in CBF from baseline to 6 months. Ischemic lesions in two participants were subtentorial and outside of the acquired ASL FOV. In the remaining participants, CBF within the infarcted tissue did not change after training (baseline: 20 ± 8; 6-month: 22 ± 9 ml·100 g^−1^·min^−1^; *P* = 0.26). In contrast to CBF, exercise training had no effect on gray matter sCoV (baseline: 50 ± 24; 6-month: 46 ± 17%; *P* = 0.31). Further, no time-by-region of interest interaction was noted (*P* = 0.99). The effect size for training on global gray matter sCoV was −0.15 (−1.22, 0.92). Based on these effect sizes, sample sizes of 29 for global CBF and 90 for sCoV are suggested to assess widespread perfusion changes in relation to exercise training.

## Discussion

This study of individuals with chronic stroke characterized the repeatability of ASL CBF, and subsequently measured changes in cerebral hemodynamics after participation in a 6-month cardiovascular rehabilitation program. Test-retest reliability for global CBF and sCoV, i.e., the spatial distribution of ASL signal across the brain, was moderate and excellent, respectively. Repeatability was greatest in the frontal lobes. Following exercise training, whole brain gray matter CBF was increased 15%. Trends for increased flow were observed in the frontal and occipital lobes; however only the parietal lobe showed a significant elevation after accounting for multiple comparisons. In contrast with the hypotheses, no change in sCoV was observed following rehabilitation, suggesting that training had a greater impact on end-stage tissue perfusion level rather than the spatial pattern of hemodynamic blood flow distribution.

While group mean interscan differences were small for both measures in the repeatability study, individual variability, as judged by ICC and within-subject coefficient of variation, was greater for CBF than sCoV. Our protocol yielded more reliable gray matter CBF estimates compared to published values from healthy older adults and those with mild cognitive impairment using a similar 2D echo planar imaging ASL acquisition (Kilroy et al., 2014). In comparison to 2D imaging, however, 3D readout with background suppression tends to have increased temporal and spatial signal-to-noise ratio (Vidorreta et al., 2013), and consequently test-retest reliability, with ICC as high as 0.93 for global gray matter CBF (Xu et al., 2010; Kilroy et al., 2014). To optimize CBF estimates, our ASL procedure incorporated an initial acquisition delay of 1600 ms prior to readout of the first slice, ensuring a mean post-label delay of ~2000 ms for the whole brain (Alsop et al., 2015). Further, we used a post-processing strategy to minimize motion and optimize GM CBF signal-to-noise ratio (Shirzadi et al., 2015). These strategies likely contributed to greater reliability than previously shown using a 2D acquisition (Kilroy et al., 2014).

The finding of elevated parietal CBF following exercise training is novel. Previous exercise trials involving healthy older adults and patients with vascular disease have reported increased CBF within the anterior cingulate (Chapman et al., 2013; Anazodo et al., 2015) and temporal cortices (Moore et al., 2015). A direct association between aerobic fitness and resting parietal CBF has been shown in chronic stroke (Robertson et al., 2015a) and Masters athletes (Thomas et al., 2013); however, the current study was not powered to determine if the change in parietal CBF was associated with the training-related increase in $\overset{\cdot }{\text{V}}{\text{O}}_{2}\text{peak}$. The only other training study in chronic stroke which measured CBF using ASL reported increased perfusion to a small region within the medial temporal lobe, but no change in global CBF (Moore et al., 2015). Notably, the volume of aerobic exercise in that study was 45 min per week, which is below the recommended volume of 60–180 min per week for aerobic training in stroke rehabilitation (Billinger et al., 2014). Participants in the current study exercised for 157 min per week by the end of the program; accumulating an exercise volume ~3.5-fold greater than participants in this previous work. The greater exercise volume in the current intervention may have contributed to a more pronounced cerebrovascular effect. Even with this greater volume of exercise training, the effect size for a change in global CBF was small. The regional finding of elevated parietal CBF is relevant from a brain health perspective given that parietal networks are implicated in executive dysfunction related to cardiovascular risk (Chuang et al., 2014). Individuals with stroke have an increased risk of developing dementia (Savva and Stephan, 2010), and parietal hypometabolism, in particular, has been linked to the progression of cognitive impairment (Drzezga et al., 2003).

A transcranial Doppler ultrasound study of stroke rehabilitation reported enhanced cerebrovascular reactivity, but no change of resting blood flow velocity, in the middle cerebral artery following 6 weeks of exercise training (Ivey et al., 2011). That study did not measure arterial diameter, so a change in blood flow despite no change in velocity cannot be ruled out. The lack of a change in velocity, however, is consistent with our finding of no change in sCoV, given the relationship between sCoV, blood flow velocity and arterial transit time (Mutsaerts et al., 2017; Robertson et al., 2017). Spatial CoV of ASL with a single post-label delay is a heuristic index of arterial transit time (Mutsaerts et al., 2017), whereby a prolonged transit time leads to greater spatial variation of intensities within the CBF image. Thus, the sCoV metric is an index of global perfusion efficiency, as mediated by conduit arteries. The null sCoV finding suggests that elevated CBF with training is unrelated to changes in large artery architecture, and instead may result from increased inflow associated with improved central cardiovascular function (e.g., increased cardiac output; Tang et al., 2014).

This study was designed as a proof-of-principle project to examine prominent cerebrovascular effects of exercise training in adults with chronic stroke. As such, the findings need to be viewed within the scope of a few limitations. First, the sample size of this study is small, reflecting its preliminary nature. Sample size estimates based on the calculated effect shown here suggest that about 30 participants may be necessary to determine the generalizability of a training effect on CBF within this patient population. Second, the repeatability sample and the observational sample may reflect individuals at different stages of stroke recovery. The comparison group, which was further post-stroke, may therefore have plateaued with respect to potential plasticity of CBF given evidence that the natural recovery of autoregulatory and neurovascular mechanisms occurs within 6 months (Cuadrado et al., 1999; Salinet et al., 2014). As such, it is unknown if exercise training would alter CBF in patients who are more than a year removed from their stroke. Moreover, the observational group might be expected to exhibit more variability in their CBF measurements, which would only be realized through a proper control group. For this preliminary study, we recruited clinical patients who had been referred to the clinical rehabilitation program* a priori* by their primary care physician and could not ethically be assigned to a control group for comparison. The existence of regional differences for the change in CBF after training, however, importantly identifies regions where the potential for exercise-based rehabilitation strategies may have the greatest impact. In a larger sample, hemispheric and even voxel-wise, differences within these lobes should be explored for greater spatial specificity of an exercise effect. A third consideration relates to the intervention which involved a combination of aerobic and resistance training. This prevents us from discerning the individual contributions of the different exercise modes; however, it does more closely reflect current models of exercise rehabilitation in the community as well as current guidelines for stroke rehabilitation (Billinger et al., 2014). Although hypothesized mechanisms linking aerobic exercise training to neuroplasticity are further developed (Constans et al., 2016), at least preliminary evidence of a positive association between resistance training and CBF has been reported (Xu et al., 2014). The differential effects of aerobic only and combination training on brain health in chronic stroke is an area of future research.

This observational study investigated the effect of a 6-month cardiovascular rehabilitation program on cerebrovascular health in individuals with chronic stroke. Gray matter CBF was increased after training, particularly within the parietal lobe; however, no change in the sCoV was noted. The parietal lobe includes a number of key communication hubs relating to executive function. Given its role in cognition, increased parietal CBF may be one mechanism through which exercise mitigates future risk of cognitive decline for people with stroke.

## Author Contributions

SM, LEM and BJM: conception and design of the research. ADR, SM, VSB and PIO: acquisition of the data. ADR and BJM: analysis and interpretation of the data. ADR: drafting of the manuscript. SM, LEM, VSB, PIO and BJM: critical revision of the manuscript. ADR, SM, LEM, VSB, PIO and BJM: final approval of the version to be published.

## Conflict of Interest Statement

The authors declare that the research was conducted in the absence of any commercial or financial relationships that could be construed as a potential conflict of interest.

## Footnotes

^1^www.fmrib.ox.ac.uk/fsl

^2^www.r-project.org

^3^www.gpower.hhu.de/en.html
